# Electrical impedance tomography compared to positron emission tomography for the measurement of regional lung ventilation: an experimental study

**DOI:** 10.1186/cc7900

**Published:** 2009-05-29

**Authors:** JC Richard, C Pouzot, A Gros, C Tourevieille, D Lebars, F Lavenne, I Frerichs, C Guérin

**Affiliations:** 1 Service de Réanimation Médicale et d'Assistance Respiratoire, Hôpital de la Croix Rousse 103 Grande Rue de la Croix Rousse, Lyon, 69004, France; 2 Creatis, Centre National de la Recherche Scientifique Unité Mixte de Recherche 5220 and Institut National de la Santé et de l'Enseignement et de la Recherche Médicale U 630, 7 avenue Jean Capelle, Villeurbanne, 69621 Cedex, France; 3 Université de Lyon, Université Claude Bernard Lyon 1, 8 avenue Rockefeller, Lyon, 69008, France; 4 Service de Soins Intensifs Animaux et Medecine d'Urgence, Ecole Nationale Vétérinaire de Lyon, 1 Avenue Bourgelat, Marcy L'Etoile, 69280, France; 5 Centre de Recherche Médicale par Emission de Positrons, Imagerie du vivant, 59 Boulevard Pinel, 69003, Lyon, France; 6 Anaesthesiology and Intensive Care Medicine, University Medical Centre Schleswig-Holstein, Kiel, Germany

## Abstract

**Introduction:**

Electrical impedance tomography (EIT), which can assess regional lung ventilation at the bedside, has never been compared with positron-emission tomography (PET), a gold-standard to quantify regional ventilation. This experiment systematically compared both techniques in injured and non-injured lungs.

**Methods:**

The study was performed in six mechanically ventilated female piglets. In normal lungs, tidal volume (V_T_) was randomly changed to 6, 8, 10 and 15 ml/kg on zero end-expiratory pressure (ZEEP), then, at V_T _10 ml/kg, positive end-expiratory pressure (PEEP) was randomly changed to 5, 10 and 15 cmH_2_O. Afterwards, acute lung injury (ALI) was subsequently created in three animals by injecting 3 ml/kg hydrochloric acid into the trachea. Then at PEEP 5 cmH_2_O, V_T _was randomly changed to 8 and 12 ml/kg and PEEP of 10 and 15 cmH_2_O applied at V_T _10 ml/kg. EIT and PET examinations were performed simultaneously. EIT ventilation (V_TEIT_) and lung volume (V_L_) were measured in the anterior and posterior area of each lung. On the same regions of interest, ventilation (V_PET_) and aerated lung volume (VA_atten_) were determined with PET.

**Results:**

On ZEEP, V_TEIT _and V_PET _significantly correlated for global (V_TEIT _= VPET - 2E-13, R^2 ^= 0.95, *P *< 0.001) and regional (V_TEIT _= 0.81V_PET_+7.65, R^2 ^= 0.63, *P *< 0.001) ventilation over both conditions. For ALI condition, corresponding R^2 ^were 0.91 and 0.73 (*P *< 0.01). Bias was = 0 and limits of agreement were -37.42 and +37.42 ml/min for global ventilation over both conditions. These values were 0.04 and -29.01 and +29.08 ml/min, respectively, for regional ventilation. Significant correlations were also found between V_L _and VA_atten _for global (V_L _= VA_atten_+1E-12, R^2 ^= 0.93, *P *< 0.0001) and regional (V_L _= 0.99VA_atten_+0.92, R^2 ^= 0.65, *P *< 0.001) volume. For ALI condition, corresponding R^2 ^were 0.94 (*P *< 0.001) and 0.54 (*P *< 0.05). Bias was = 0 and limits of agreement ranged -38.16 and +38.16 ml for global ventilation over both conditions. These values were -0.24 and -31.96 to +31.48 ml, respectively, for regional ventilation.

**Conclusions:**

Regional lung ventilation and volume were accurately measured with EIT in healthy and injured lungs and validated by simultaneous PET imaging.

## Introduction

Electrical impedance tomography (EIT) is a new lung imaging modality. It might become highly relevant to managing patients with acute respiratory distress syndrome (ARDS) in the intensive care unit (ICU) because it can estimate regional lung ventilation at the bedside [1]. An acceptable agreement, namely bias of 0% and limits of agreement of -10 to +10%, has been found between EIT and computed tomography (CT) in detecting right-to-left lung changes in gas volume [2]. However, x-ray CT does not measure lung ventilation directly. Concerns were raised about the ability of EIT to accurately quantify ventilation in an experimental study using single photon emission computed tomography (SPECT) as a reference [3]. However, whether the slight disagreement between the two methods is attributed to EIT or SPECT remains unknown. Positron emission tomography (PET) is a non-invasive and powerful method to quantify alveolar ventilation and volume [4], and alveolar recruitment [5] regionally, and may be considered as a gold standard to quantify regional lung ventilation. No study has compared both techniques and their ability to measure alveolar ventilation and volume so far. Furthermore, the capability of EIT to detect changes over a large range of end expiratory lung volume and delivered tidal volume (V_T_) has only seldom been studied so far. Therefore, the primary goal of the present study was to compare EIT with PET after changing lung ventilation and volume in anesthetized pigs.

## Materials and methods

### Animals

The protocol was approved by our Institutional Review Board for the care of animal subjects. The care and handling of the animals were performed in accordance with the National Institutes of Health guidelines for ethical animal research.

Six female piglets (mean ± standard deviation (SD) = 28 ± 3 kg; Table 1) were premedicated with an intramuscular injection of xylazine (20 mg), droperidol (10 mg), and ketamine (500 mg). The animals were tracheotomized and mechanically ventilated (Avea; Viasys Healthcare, Höchberg, Germany) in volume-controlled mode using V_T _10 ml/kg, fraction of inspired oxygen (FiO_2_) 0.21 during the part of the experiment on non-injured lungs, and zero end-expiratory pressure (ZEEP) (Table 1). Right internal jugular vein and carotid artery were cannulated. Anesthesia-analgesia was maintained with intravenous infusion of propofol 200 to 300 mg/hour and fentanyl 2 to 4 mcg/kg/min, and paralysis with pancuronium bromide 3 mg/hour.

**Table 1:** Baseline ventilatory settings of six pigs

Pig number	Weight (kg)	V_T_ (mL)	Rf (breaths.min)	V' (L/s)	PEEPt (cmH_2_O)	Pplat (cmH_2_O)	PaO_2 _* (mmHg)	PaCO_2 _* (mmHg)	pH*	MAP (mmHg)
1	31	310	18	0.28	0.7	11.4	100	37	7.43	85
2	30	300	20	0.30	0.0	16.0	85	38	7.44	84
3	24	250	26	0.36	0.0	15.0	80	35	7.38	86
4	30	300	17	0.28	0.0	14.0	122	28	7.53	90
5	26	260	20	0.26	0.0	8.5	124	36	7.41	69
6	30	270	23	0.35	0.3	14.0	101	37	7.42	89

Mean	28	282	21	0.31	0.17	13.2	102	35	7.44	84
SD	3	25	3	0.04	0.29	2.7	18	4	0.05	8

* inspiratory oxygen fraction was 21%

MAP = mean systemic arterial blood pressure; PEEPt = total positive end-expiratory pressure; Pplat = plateau pressure; Rf = respiratory frequency; V'= inflation flow; V_T _= tidal volume.

### Equipment

The experiments were carried out in the experimental research imaging facility of the University of Lyon (CERMEP, Lyon, France).

The EIT device used was the Goettingen Goe-MF II System (Viasys Healthcare, Höchberg, Germany). A single array of 16 electrodes (Blue Sensor, BR-80-K, AMBU, Denmark) was placed on the mid-chest circumference of the animal. Electrical currents (50 kHz, 5 mA) were injected through adjacent pairs of electrodes in a rotating mode. During each electrical current injection, the resulting potential differences were measured at adjacent electrodes pairs and the resulting impedance (Z) distribution was calculated. The EIT recordings were sampled at a rate of 13 Hz, that is, 13 scans/second.

The PET study was performed using an ECAT EXACT HR+ scanner (Siemens, CTI, Knoxville, Tennesse, USA).

Piezoresistive pressure transducers (Gabarith 682002, Becton Dickinson, Sandy, UT, USA) were calibrated at the mid-chest level and connected to a A/D card (MP 100; Biopac Systems, Santa Barbara, CA, USA). Systemic arterial blood pressure, airway pressure and airflow (Fleish 2, Lausanne, Switzerland) were continuously recorded, sampled at 200 Hz, and analyzed with Acknowledge software (Biopac MP100 Systems, Santa Barbara, CA, USA). The value of V_T _was obtained from the numerical integration of the airflow signal.

### Protocol

**Figure 1:**
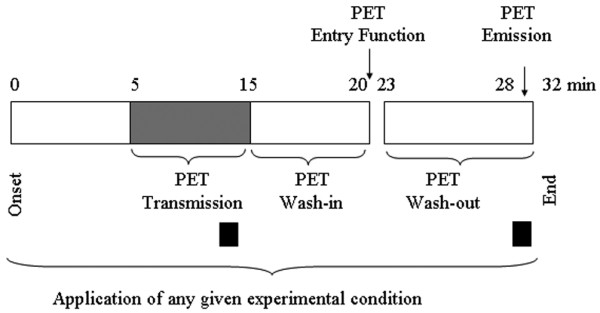
Description of one given experimental condition. During the first five minutes the experimental step, either change in tidal volume or positive end-expiratory pressure (PEEP), is applied without any measurement and continued up to the end of this phase. Then positron emission tomography (PET) transmission scan is taken for 10 minutes followed by a five-minute wash-in phase. Afterwards, ^13^N-N_2 _positron-emitting tracer is washed-out for five minutes. In-between the amount of the tracer entering the lung is measured (entry function). PET emission scans are then performed at tracer equilibrium and during tracer wash-out. The electrical impedance tomography signals used in present analysis are recorded for one minute at the end of both transmission and emission periods (black squares). Each step lasts 30 minutes.

Once preparation was completed the animal was installed into the PET camera in a supine position. Two sets of experiments were performed in each animal. First, from its baseline value of 10 ml/kg, V_T _was randomly changed to 6, 8, and 15 ml/kg on ZEEP. Second, while V_T _was kept constant at 10 ml/kg, positive end-expiratory pressure (PEEP) was randomly changed from 5 to 15 cmH_2_O by a 5 cmH_2_O-step procedure. Each step was applied for five minutes (Figure 1).

In three animals, acute lung injury (ALI) was subsequently created by injecting 3 ml/kg hydrochloric acid 0.1 M via the endotracheal tube, after having increased FiO_2 _to 100%. The target was to obtain partial pressure of arterial oxygen (PaO_2_) less than 300 mmHg 10 minutes after inhalation. Additional doses of 1 ml/kg each were allowed to be used to reach this objective. Reinjection of HCl was needed once in only one animal. Once the target was reached, PEEP was set to 3 cmH_2_O for two hours to obtain stabilization. At the end of the stabilization period, two sets of experiments were performed. First, at PEEP 5 cmH_2_O, V_T _was randomly changed to 8 and 12 ml/kg for 10 minutes each from the baseline of 10 ml/kg. Second, PEEP of 10 and 15 cmH_2_O were applied in a random order for 10 minutes, at V_T _10 ml/kg. The respiratory rate was titrated to keep arterial pH above 7.20 and intrinsic PEEP lower than 1 cmH_2_O.

Arterial blood gas was obtained from 2 ml of arterial blood injected into a cartridge (BG Cartridge, Gamida, Eaubonne, France) for immediate pH, partial pressure of carbon dioxide (PCO_2_) and partial pressure of oxygen (PO_2_) analysis using blood gas analyzer (IRMA Trupoint™, ITC, Edison, NJ, USA). At the end of each step, the following measures were assessed in this order: mean systemic arterial blood pressure; total PEEP (PEEPt) and end-inspiratory elastic recoil pressure of the respiratory system (Pplat, rs) by occluding the airways at the end of expiration for three seconds and at the end of the immediately following inspiration for four seconds, respectively; and lung ventilation.

### Assessment of regional ventilation with EIT and PET

The EIT signals were recorded continuously from the onset to the end of each experimental condition. PET assessment of ventilation was performed as follows (Figure 1). First, a transmission scan was made within 10 minutes. Then, the ^13^N-N_2 _tracer continuously produced by the cyclotron fed the ventilator and was washed-in into the lungs through the endotracheal tube, and administered synchronously with the mechanical insufflations from the activation of an electronic valve [4]. Once the activity of the tracer monitored from the camera screen plateaued, entry function of the tracer, that is, the amount of activity entering the lung, was measured at the endotracheal tube and equilibrium PET images were taken for three minutes. Then, the administration of the tracer was stopped at the very onset of inspiration and the tracer was washed-out from the lungs. Emission scans were taken for four minutes from the onset of washout to measure the tracer activity inside the lung.

### Data analysis

The EIT signals retained in the comparison with the PET data were acquired for one minute at the time of transmission scan before tracer inhalation and during the wash-out period synchronously with emission scan (black squares in Figure 1). The wash-out period was selected because the modeling of the tracer kinetic with PET was performed from the data collected during the wash-out phase. The transmission frame was used to compare the effect of PEEP on lung volume while the emission frame was selected to compare the effect of changing V_T _on lung ventilation. Therefore, this design has the unique feature of allowing the comparison between EIT and PET methods at the same time. To make the comparison between EIT and PET as accurate as possible, one of the most difficult issues to deal with was to match the same lung regions of interest (ROI) with each of the two techniques. An approximately 5 cm lung height was sampled with the 16-electrodes array [6]. We selected as closely as possible the corresponding PET planes as follows. PET field of view was defined by laser projection onto the pig's thorax. Camera bed was then positioned so that the EIT electrodes were located at PET mid-field of view. The information contained in seven contiguous PET slices located at mid-field of view was then averaged, assuring an acceptable match between regions studied with both imaging techniques.

The investigators in charge of EIT (IF) and PET (JCR) analyses were blinded to the definition of each condition and, moreover, analyzed the data independently.

EIT scans were generated using the weighted backprojection reconstruction procedure along equipotential lines [7]. EIT data was evaluated offline in terms of tidal volume (V_TEIT_) and change in lung volume (V_L_) in four ROIs corresponding to the anterior and posterior area of the right and left lungs, respectively. V_L _reflected the shift in lung mid-capacity with PEEP relative to ZEEP [8].

ROIs were drawn around both lungs using PET transmission scans, on seven contiguous tomographic slices encompassing 5.1 cm of lung height. Lung volume measured with PET from density obtained on the transmission scan (VA_atten_) was obtained from voxel-by-voxel values of lung attenuation in these ROIs, as previously described [5]. ROIs were then superimposed on PET equilibrium and wash-out scans, and voxel-by-voxel time-activity curves were analyzed as previously described using a single compartment model [4]. The modeling analysis enabled the determination of alveolar ventilation (V) expressed as ml/min/100 ml V_L _and alveolar volume. Global analyses were performed on the whole set of voxels, while regional values were computed in four quadrants corresponding to the anterior and posterior area of the right and left lungs, respectively. In each of these regions, VA_atten _and V_PET _were computed as follows:

(1)

(2)

where i refers to the i^th ^voxel of the region and n to the total number of voxels of the corresponding region.

### Statistical analysis

The values are presented as their mean ± SD. The relationships of V_TEIT _(arbitrary units, a.u.) to V_PET _(ml/min), in the first part of the experiment, were performed over the whole lungs from linear regression [9]. Then, in each quadrant, the values of V_TEIT _were computed as ml/min by using the following equation:

(3)

The same approach was used to compare VA_atten _to V_L _in the part of the study performed at different PEEP levels. The resulting predicted values of V_TEIT _and V_L _were henceforth expressed as ml/min and ml, respectively. Furthermore, since, by definition, V_L _was 0 at ZEEP, the differences in VA_atten _(ΔVA_atten_) relative to ZEEP in normal condition and to PEEP of 5 cmH_2_O in ALI condition were compared with the corresponding values of V_L _across the PEEP levels.

Linear regression was performed by using the least square method. Bias and agreement were assessed from the Bland and Altman representation [10]. The non-uniformity distribution of errors in regional measurements was checked by inspecting plots of residuals vs. predicted values. The statistical analysis was performed using SPSS statistical software (version 15.0 for Windows, SPPS Inc., Chicago, IL, USA). *P *< 0.05 was taken as the statistically significant threshold.

## Results

For technical reasons, PET images in the PEEP trial in pig number 2 and of V_T _10 ml/kg on ZEEP in pig number 4 were not available. Therefore, in this pig ΔVA_atten _could not be computed. Moreover, pig number 6 did not experience V_T _8 ml/kg in the ALI condition. Therefore, 23 normal conditions and 8 ALI conditions were available for the data analysis.

### Effects of changing V_T _at ZEEP on ventilation

**Figure 2:**
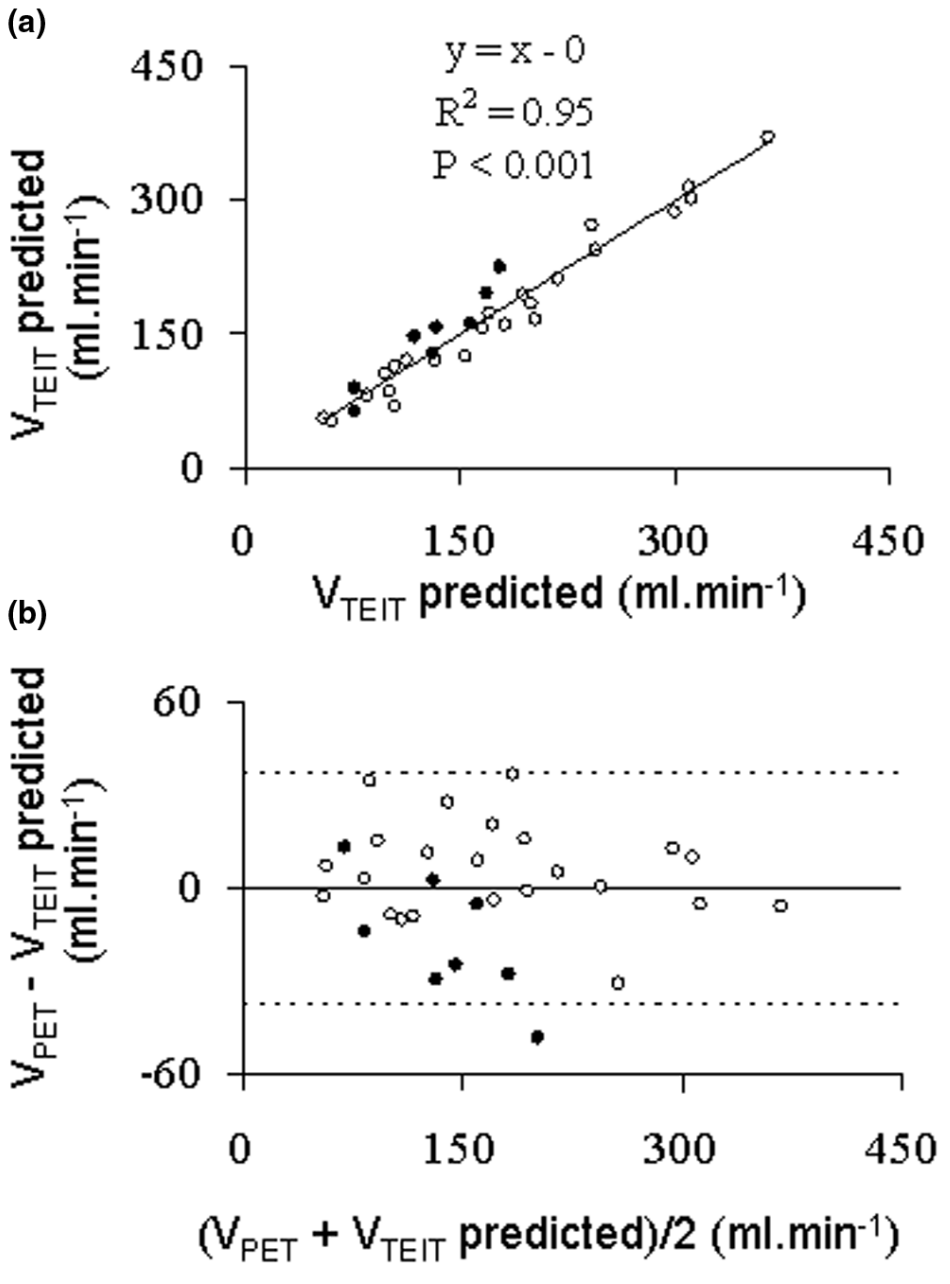
Global lung ventilation. **(a) **Relationship of global lung ventilation measured with electrical impedance tomography (V_TEIT predicted_) and positron emission tomography (V_PET_) in the first part of the experiment. The regression line was drawn over all experimental points pertaining to both normal (open circles) and acute lung injury (closed circles) conditions. **(b) **Relationship of the difference to the mean of global lung ventilation measured with electrical impedance tomography (V_TEIT predicted_) and positron emission tomography (V_PET_) in the first part of the experiment. Horizontal continuous line and horizontal broken lines are the mean and the upper (mean + 2 standard deviations) and lower (mean - 2 standard deviations) values of the difference, respectively.

**Figure 3:**
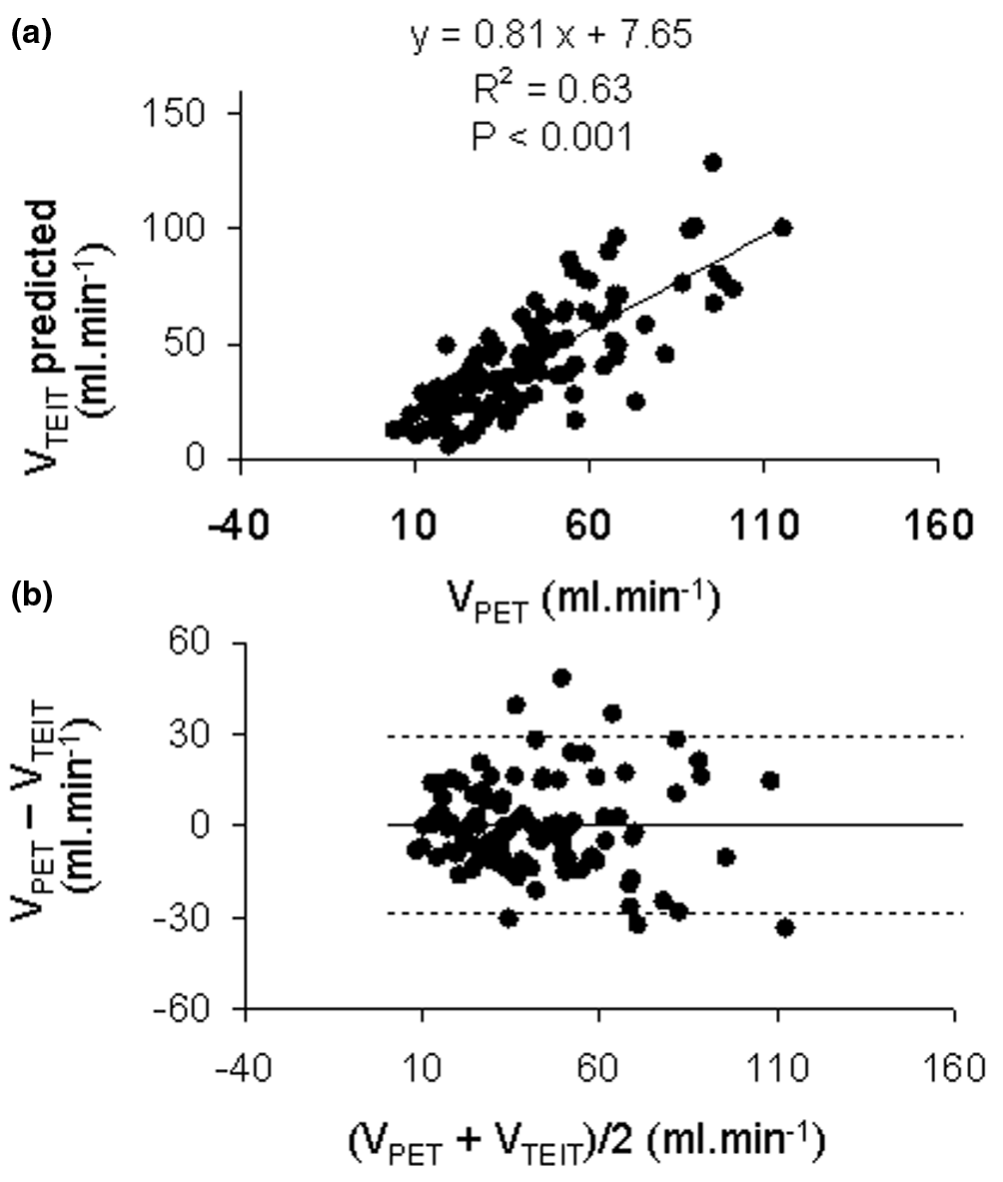
Regional Lung Ventilation. **(a) **Relationship of regional lung ventilation measured with electrical impedance tomography (V_TEIT predicted_) and positron emission tomography (V_PET_) in the first part of the experiment. The regression line was drawn over all experimental points pertaining to normal and acute lung injury conditions in each quadrant. **(b) **Relationship of the difference to the mean of regional lung ventilation measured with electrical impedance tomography (V_TEIT predicted_) and positron emission tomography (V_PET_) in the first part of the experiment. Horizontal continuous line and horizontal broken lines are the mean and the upper (mean + 2 standard deviations) and lower (mean - 2 standard deviations) values of the difference, respectively.

We found a strong correlation between global V_TEIT _and V_PET _(Figure 2a) over both conditions. The coefficients of determination were 0.95 and 0.91 (*P *< 0.001) in normal and ALI conditions, respectively. There were no bias and narrow limits of agreement (-37.42 to +37.42 ml/min) over both conditions (Figure 2b). The bias amounted to 5.77 and limits of agreement -24.49 to +36.03 ml/min for normal condition, and -16.59 and -55.26 to +22.08 ml/min for ALI condition. For regional ventilation, the correlation was slightly weaker but still significant (Figure 3a) over both conditions. The coefficients of determination were 0.63 in normal condition and 0.73 in ALI condition (*P *< 0.01). There were no fixed bias and narrow limits of agreement (-29.01 to +29.08 ml/min) over both conditions (Figure 3b). The bias was 1.47 and limits of agreement -29.71 to +32.66 ml/min for the normal condition, and 0.91 and -27.94 to +29.76 ml/min for ALI.

### Effects of PEEP on lung volume

**Figure 4:**
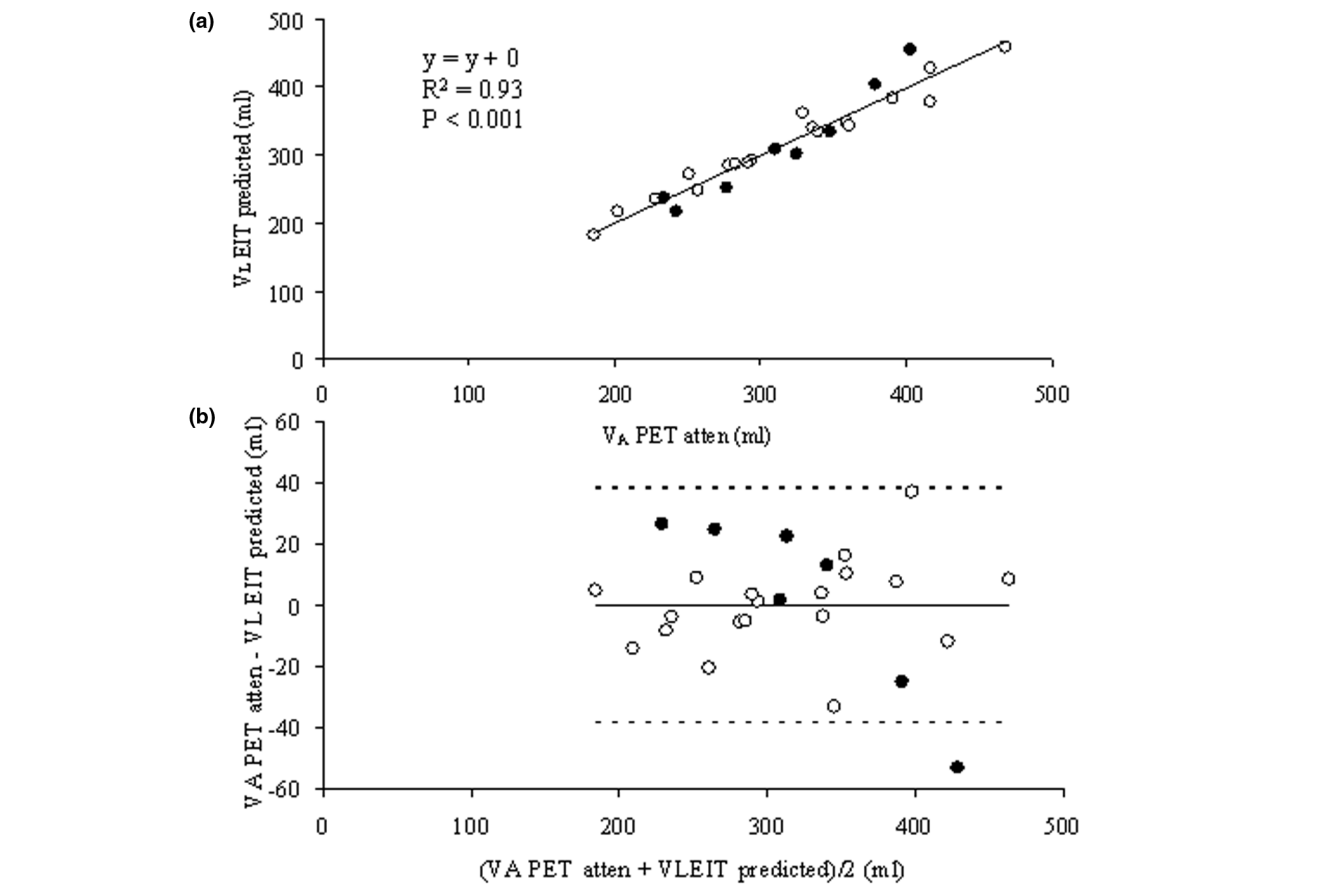
Global lung volume. **(a) **Relationship of global lung volume measured with electrical impedance tomography (V_LEIT predicted_) and positron emission tomography (VA_atten_) in the second part of the experiment. The regression line was drawn over all experimental points pertaining to both normal (open circles) and acute lung injury (closed circles) conditions. **(b) **Relationship of the difference to the mean of global lung volume measured with electrical impedance tomography (V_LEIT predicted_) and positron emission tomography (VA_atten_) in the second part of the experiment. Horizontal continuous line and horizontal broken lines are the mean and the upper (mean + 2 standard deviations) and lower (mean - 2 standard deviations) values of the difference, respectively.

**Figure 5:**
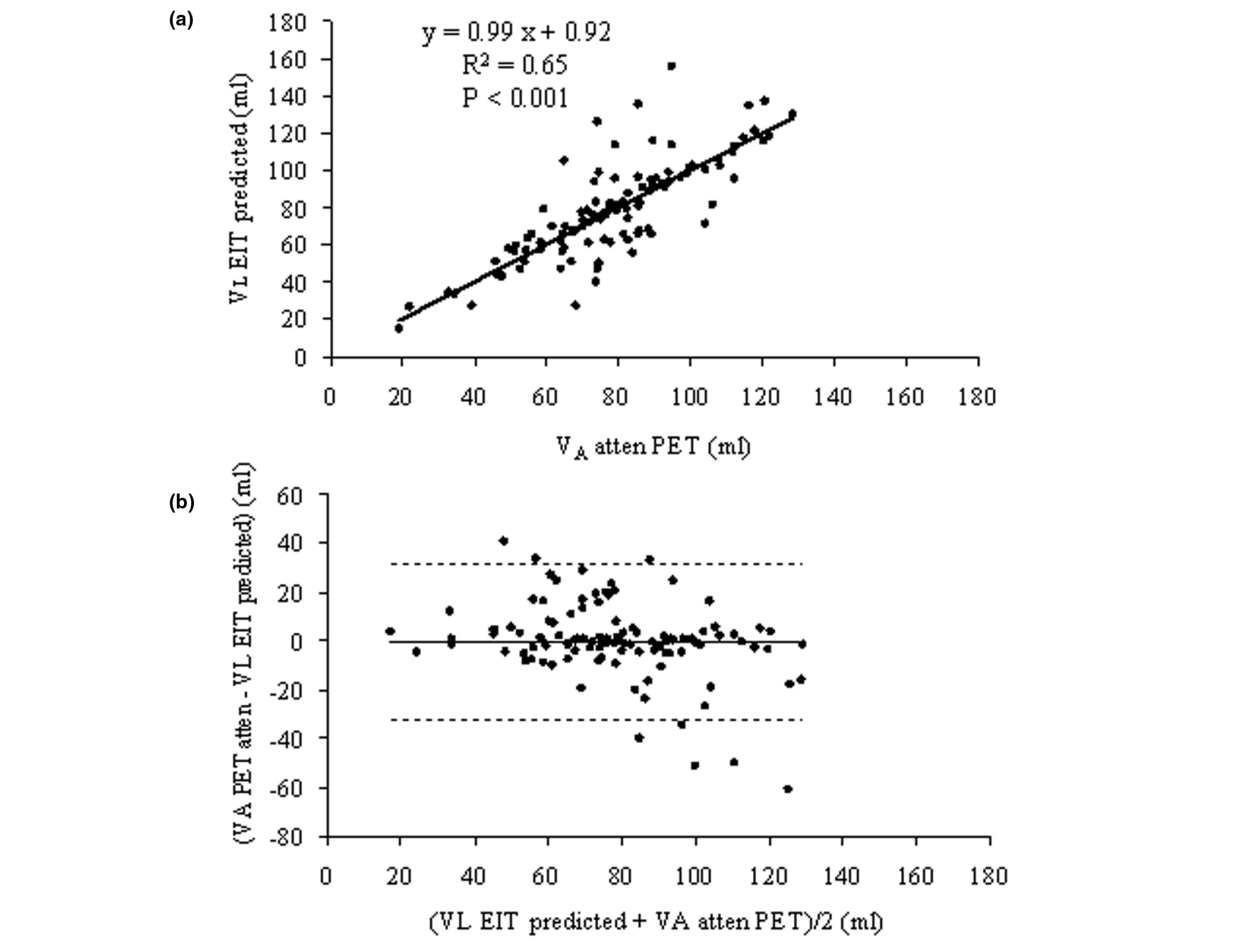
Regional lung volume.  **(a) **Relationship of regional lung volume measured with electrical impedance tomography (V_LEIT predicted_) and positron emission tomography (VA_atten_) in the second part of the experiment. The regression line was drawn over all experimental points pertaining to normal and acute lung injury conditions in each quadrant. **(b) **Relationship of the difference to the mean of regional lung volume measured with electrical impedance tomography (V_LEIT predicted_) and positron emission tomography (VA_atten_) in the second part of the experiment. Horizontal continuous line and horizontal broken lines are the mean and the upper (mean + 2 standard deviations) and lower (mean - 2 standard deviations) values of the difference, respectively.

We found a strong correlation between global VA_atten _and V_L _over both conditions (Figure 4a). The coefficients of determination were 0.96 and 0.94 (*P *< 0.001) for normal and ALI, respectively. There were no bias and acceptable limits of agreement (-38.16 to +38.16 ml) over both conditions (Figure 4b). The bias (limits of agreement) were 0.28 (-30.17 to +29.61) ml for normal condition and 0.62 (-51.53 to +52.78) ml for ALI. At the regional level, the correlation was lower but still significant over both conditions (Figure 5a). The coefficients of determination were 0.76 (*P *< 0.01) and 0.54 (*P *< 0.05) for normal and ALI, respectively. There was no bias and limits of agreement ranged from -31.96 to +31.48 ml over both conditions. The bias (limits of agreement) were 0.21 (-26.17 to +26.58) ml for normal condition and -2.54 (-41.88 to +36.80) ml for ALI. The results pertaining to ΔVA_atten _instead of VA_atten _were similar (not shown).

**Figure 6:**
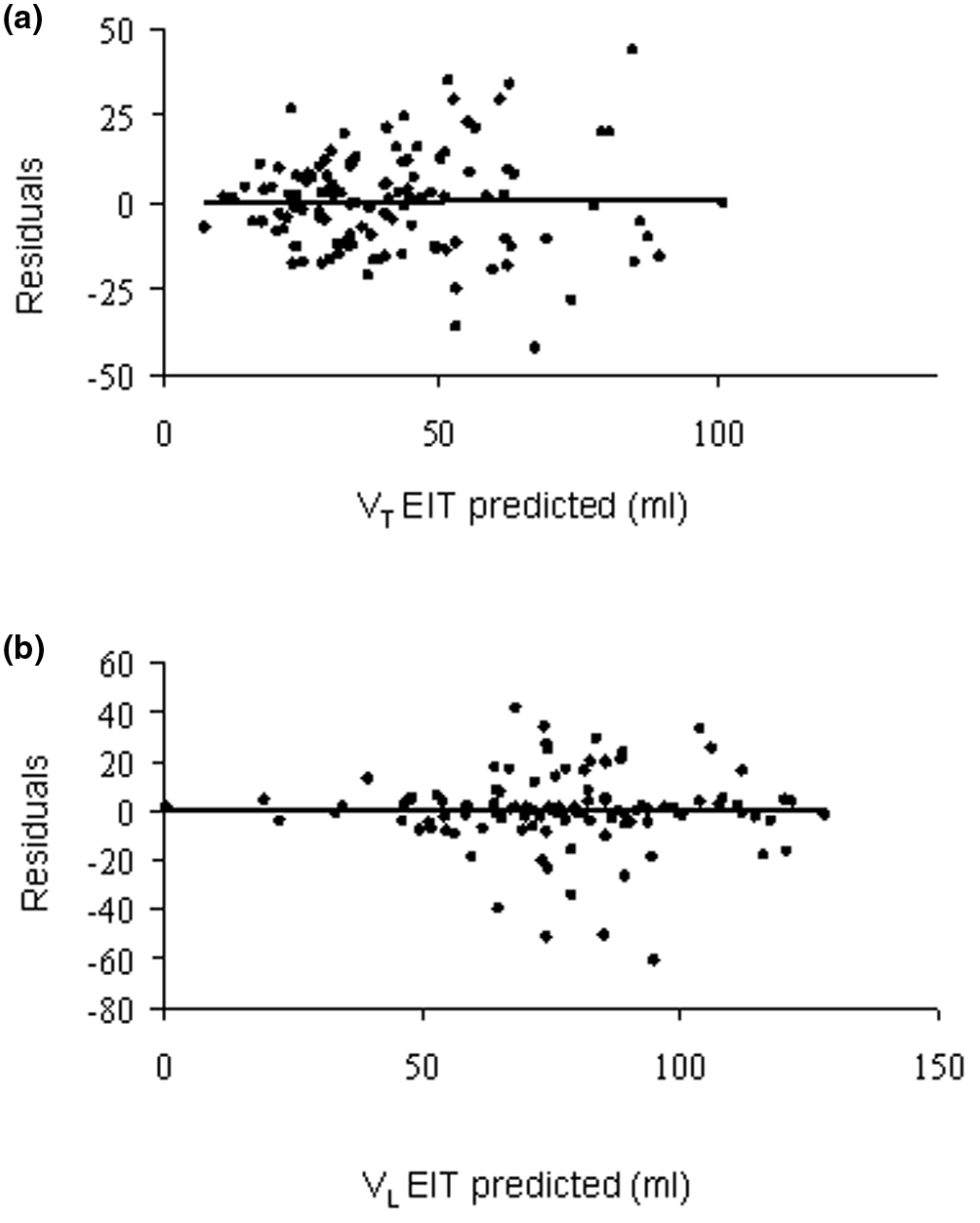
Plots of the residuals to the predicted values. **(a) **Regional ventilation (V_TEIT_) and **(b) **volume (V_L _EIT).

Inspection of plots of residuals vs. predicted values disclosed that errors in measurements were uniformly distributed (Figure 6).

## Discussion

The present study showed that the measurement of lung ventilation and volume with EIT compared favourably with PET assessment. In contrast to previous validation studies using established lung imaging modalities, it must be stressed that in our present study the comparison between the two techniques was performed at the same time. Therefore, lung ventilation and volume were assessed with the same ventilatory history.

EIT could be an important tool in the future because it might allow the intensivist to monitor the regional lung ventilation and volume at the bedside in ICU patients and to manage ventilatory settings on this basis. Therefore, the validity of the measurements obtained with EIT is crucial. PET is a gold standard to quantify lung ventilation on a regional basis. Hinz and colleagues, in a porcine model of oleic acid-induced lung injury, compared SPECT and EIT [3] to measure lung ventilation. The linear relationship between regional ventilation measured with SPECT and EIT, both expressed in percentage of total ventilation, had a slope of 0.82, an intercept of 0.73, and R^2 ^of 0.92. Although the slope of the relationship of regional ventilation with both techniques was identical in the two studies, the values of R^2 ^were lower in our study. Indeed, the regional points were scattered as shown on Figure 3a. In the study by Hinz and colleagues [3], the Bland Altmann plots of the ventilation expressed in percentage clearly indicated a proportional bias with the slopes of the linear relationships drawn over the experimental points of the difference to the mean different from 0. This was not the case in our study, which was unbiased.

Apart from non-spatial coincidence in the ROIs drawn with each technique, which is a potential flaw in any such validation studies, two reasons for lower R^2 ^in our study may be raised. First, the present study was performed on ZEEP, so ventilation heterogeneity across quadrants should be expected in connection with anesthesia-related atelectasis. On the other hand, PEEP 5 cmH_2_O in the study by Hinz and colleagues [3] may have homogenized lung ventilation in the easily recruitable model of oleic acid-induced ALI. Ventilation heterogeneity is expected to increase errors related to spatial coincidence between techniques and may have jeopardized the results in the present study. Second, unlike the study by Hinz and colleagues [3], we applied a wide range of V_T_. This may have challenged EIT validity to assess lung ventilation, because lung water and blood redistribution induced by V_T _change may affect the EIT signal.

Frerichs and colleagues compared the measurements of aerated lung volume with EIT and electron beam CT [11] and found significant correlations between the two methods. Significant correlations were also obtained between EIT and CT scan by Victorino and colleagues [2] in ARDS patients. More recently, Wrigge and colleagues simultaneously compared CT scan and EIT in pigs whose lungs were injured by acid aspiration or oleic acid plus abdominal hypertension [12] and found that both techniques were highly correlated (R^2 ^= 0.63 to 0.88, *P *< 0.0001, bias <5%) in both injuries. The variability between methods was lower in direct than indirect ALI.

In the present study the values of lung ventilation and volume measured with EIT have been quantified and expressed as ml/min and ml, respectively, and not as arbitrary units. This attempt at quantification is a relevant approach because results can be compared between patients and are more meaningful in the clinical field.

Our study has limitations such as the small number of animals investigated. Moreover, the low spatial resolution of EIT renders a more detailed regional analysis difficult. This is a reason why we did not carry out a pixel-by-pixel analysis over ROIs drawn along a ventral-to-dorsal axis. This latter analysis is, however, being investigated further in our laboratory. Furthermore, ventilation and lung volume measurements with PET have methodological limitations. Briefly, partial-volume averaging and spill-over effects affect radioactivity quantification with PET, mainly in the peripheral parts of the lungs. Furthermore, modelling ^13^N kinetics requires several assumptions that are simplification of such a complex physiologic processes such as alveolar ventilation [4]. Nevertheless, PET is an accurate and unbiased tool to quantify alveolar ventilation and lung volume [4]. Finally, the animals were not ventilated in such a way as to prevent VILI (Ventilator-Induced Lung Injury). However, this was not a disadvantage in the present design as it allowed us to compare the EIT and PET findings even with a non-optimized ventilation strategy.

One of the strengths of this study is that EIT was tested during conditions in which its validity was really challenged. As stated above, despite PEEP and V_T _variation over a wide range of values, EIT measurements remained acceptably correlated with PET at the regional level. This favors the use of EIT in the clinical setting to test the effect of different PEEP levels or recruiting maneuvers. It should be noted that PEEP is not a recruitment maneuver *per se*, but an appropriate tool to keep the lung open after an adequate and individualized recruitment procedure.

### Clinical implications

EIT analysis could be refined and extended further by implementing pixel-by-pixel analysis and by better defining atelectasis, so the functional lung recruitment should be assessed. Indeed, the lung recruitability [13] measured with the CT scan are anatomic features. However, for the lung mass recruited to be a relevant issue it should correspond to an increase in ventilation in those areas which continue to receive blood flow and, hence, should contribute to reduce the functional shunt. It has recently been shown that anatomic shunt and functional shunt do not correlate in ARDS patients [14]. As lung perfusion could be assessed with EIT [15], this tool should be well suited to deal with these key issues. Further studies would be welcome to address these questions.

## Conclusions

We found that regional lung ventilation and volume were accurately measured with EIT by using PET as the validation tool, over a wide range of PEEP and V_T_.

## Key messages

• In normal and injured pig lungs EIT accurately measures regional lung ventilation.

• This result is obtained from comparison with PET, which is the gold standard to quantify the regional lung ventilation.

## Abbreviations

ALI: acute lung injury; ARDS: acute respiratory distress syndrome; CT: computed tomography; ΔZ: change in thorax electrical impedance; EIT: electrical impedance tomography; FiO_2_: fraction of inspired oxygen; ICU: intensive care unit; PaO_2_: partial pressure of arterial oxygen; PCO_2_: partial pressure of carbon dioxide; PEEP: positive end-expiratory pressure; PEEPt: total positive end-expiratory pressure; PET: positron emission tomography; PO_2_: partial pressure of oxygen; ROI: region of interest; SD: standard deviation; SPECT: single photon emission computed tomography; VAatten: lung volume measured with PET from density obtained on the transmission scan; VILI: Ventilator-Induced Lung Injury; V_L_: change in lung mid-capacity measured with EIT; V_PET_: lung ventilation measured from PET emission scan; V_T_: tidal volume delivered by the ventilator; V_TEIT_: tidal volume measured with EIT; Z: impedance; ZEEP: zero end-expiratory pressure.

## Competing interests

CardinalHealth provided a grant to support the study. These fundings were not used to finance the manuscript. The manuscript was financed by academic funds from the authors' laboratory. The authors declare no other competing interests.

## Authors' contributions

JCR participated in the design of the study and in all experiments, analyzed the PET data and drafted the paper. CP participated in all experiments and in the PET data analysis. AG participated in all experiments and in the PET data analysis. CT participated in all experiments and provided us with tracers administration. DL participated in all experiments and provided us with tracers administration. FL participated in all experiments and provided us with PET data acquisition. IF participated in the design of the study and initial experiments, analyzed the EIT data and drafted the paper. CG participated in the design of the study and in all experiments, performed the data analysis, and drafted the paper.

## Authors' information

JCR is associate professor of critical care medicine and research director. CP was a research fellow during this experiment. AG was a research fellow during this experiment. CT is a technician in charge of the chemistry in the platform. DL is a pharmacist in charge of the chemistry in the platform. FL is an engineer in charge of the PET camera. IF is a professor of physiology and was a visiting professor at the time of this experiment. CG is a professor of critical care medicine and research director.

## Note

This work has been performed at the CERMEP Imagerie du vivant, 59 Boulevard Pinel, 69677 Bron Cedex, France.

## References

[B1] Frerichs I, Dargaville PA, Dudykevych T, Rimensberger PC (2003). Electrical impedance tomography: a method for monitoring regional lung aeration and tidal volume distribution?. Intensive Care Med.

[B2] Victorino JA, Borges JB, Okamoto VN, Matos GF, Tucci MR, Caramez MP, Tanaka H, Sipmann FS, Santos DC, Barbas CS, Carvalho CR, Amato MB (2004). Imbalances in regional lung ventilation: a validation study on electrical impedance tomography. Am J Respir Crit Care Med.

[B3] Hinz J, Neumann P, Dudykevych T, Andersson LG, Wrigge H, Burchardi H, Hedenstierna G (2003). Regional ventilation by electrical impedance tomography: a comparison with ventilation scintigraphy in pigs. Chest.

[B4] Richard JC, Janier M, Lavenne F, Tourvieille C, Le Bars D, Costes N, Gimenez G, Guerin C (2005). Quantitative assessment of regional alveolar ventilation and gas volume using 13N-N2 washout and PET. J Nucl Med.

[B5] Richard JC, Le Bars D, Costes N, Bregeon F, Tourvieille C, Lavenne F, Janier M, Gimenez G, Guerin C (2006). Alveolar recruitment assessed by positron emission tomography during experimental acute lung injury. Intensive Care Med.

[B6] Rabbani KS, Hassan M, Kiber A (1996). 3D object localization using EIT measurements at two levels. Physiol Meas.

[B7] Barber DC (1990). Quantification in impedance imaging. Clin Phys Physiol Meas.

[B8] Richard JC, Bregeon F, Costes N, Bars DL, Tourvieille C, Lavenne F, Janier M, Bourdin G, Gimenez G, Guerin C (2008). Effects of prone position and positive end-expiratory pressure on lung perfusion and ventilation. Crit Care Med.

[B9] Meier T, Luepschen H, Karsten J, Leibecke T, Grossherr M, Gehring H, Leonhardt S (2008). Assessment of regional lung recruitment and derecruitment during a PEEP trial based on electrical impedance tomography. Intensive Care Med.

[B10] Bland JM, Altman DG (1986). Statistical methods for assessing agreement between two methods of clincial measurement. Lancet.

[B11] Frerichs I, Hinz J, Herrmann P, Weisser G, Hahn G, Dudykevych T, Quintel M, Hellige G (2002). Detection of local lung air content by electrical impedance tomography compared with electron beam CT. J Appl Physiol.

[B12] Wrigge H, Zinserling J, Muders T, Varelmann D, Gunther U, Groeben C von der, Magnusson A, Hedenstierna G, Putensen C (2008). Electrical impedance tomography compared with thoracic computed tomography during a slow inflation maneuver in experimental models of lung injury. Crit Care Med.

[B13] Gattinoni L, Caironi P, Cressoni M, Chiumello D, Ranieri VM, Quintel M, Russo S, Patroniti N, Cornejo R, Bugedo G (2006). Lung recruitment in patients with the acute respiratory distress syndrome. N Engl J Med.

[B14] Cressoni M, Caironi P, Polli F, Carlesso E, Chiumello D, Cadringher P, Quintel M, Ranieri VM, Bugedo G, Gattinoni L (2008). Anatomical and functional intrapulmonary shunt in acute respiratory distress syndrome. Crit Care Med.

[B15] Frerichs I, Hinz J, Herrmann P, Weisser G, Hahn G, Quintel M, Hellige G (2002). Regional lung perfusion as determined by electrical impedance tomography in comparison with electron beam CT imaging. IEEE Trans Med Imaging.

